# CCN3 and DLL1 co-regulate osteogenic differentiation of mouse embryonic fibroblasts in a Hey1-dependent manner

**DOI:** 10.1038/s41419-018-1234-1

**Published:** 2018-12-11

**Authors:** Xin Su, Yalin Wei, Junjie Cao, Xiulin Wu, Daiyong Mou, Jinyong Luo, Aifang Li, Guo-Wei Zuo, Min Tang

**Affiliations:** 1 Key Laboratory of Diagnostic Medicine designated by the Chinese Ministry of Education, Chongqing Medical University, 400016 Chongqing, China; 2 Xi’an No.4 Hospital, 710004 Xi’an, China; 3 Department of Clinical Laboratory, Xi’an No.5 Hospital, 710082 Xi’an, China; 4 Department of Geriatrics, Army Military Medical University, 400038 Chongqing, China; 5 Department of Clinical Laboratory, Xi’an Chest Hospital, 710100 Xi’an, China

## Abstract

Notch signaling pathway is one of the most important pathways to regulate intercellular signal transduction and is crucial in the regulation of bone regeneration. Nephroblastoma overexpressed (NOV or CCN3) serves as a non-canonical secreted ligand of Notch signaling pathway and its role in the process of osteogenic differentiation of mesenchymal stem cells (MSCs) was undefined. Here we conducted a comprehensive study on this issue. In vivo and in vitro studies have shown that CCN3 significantly inhibited the early and late osteogenic differentiation of mouse embryonic fibroblasts (MEFs), the expression of osteogenesis-related factors, and the subcutaneous ectopic osteogenesis of MEFs in nude mice. In mechanism studies, we found that CCN3 significantly inhibited the expression of BMP9 and the activation of BMP/Smad and BMP/MAPK signaling pathways. There was also a mutual inhibition between CCN3 and DLL1, one of the classic membrane protein ligands of Notch signaling pathway. Additionally, we further found that Hey1, the target gene shared by BMP and Notch signaling pathways, partially reversed the inhibitory effect of CCN3 on osteoblastic differentiation of MEFs. In summary, our findings suggested that CCN3 significantly inhibited the osteogenic differentiation of MEFs. The inhibitory effect of CCN3 was mainly through the inhibition of BMP signaling and the mutual inhibition with DLL1, so as to inhibit the expression of Hey1, the target gene shared by BMP and Notch signaling pathways.

## Introduction

Notch signaling pathway is one of the highly conserved signaling pathways in the process of biological evolution^1^. In the process of mammalian growth and cell differentiation, proliferation, and apoptosis, Notch signaling pathway plays an irreplaceable role^2^. It is also crucial in the process of bone regeneration^3–6^. The ligands (DLL1, DLL3, DLL4, Jagged1, and Jagged2) and receptors (Notch1, Notch2, Notch3, and Notch4) of canonical Notch signaling pathway are cell membrane proteins that can interact to activate Notch signaling pathway only when cells adhere to each other^7,8^, thus regulating the expression of the target genes, such as Hey1^2,9–12^. The current study found that, in addition to the canonical membrane protein ligands, there is also a secreted ligand for the Notch signaling pathway: nephroblastoma overexpressed (NOV/CCN3). It can affect the Notch signaling pathway when cells do not adhere to each other^13–15^. CCN3 is a member of the CCN family and is widely expressed in the nervous system, musculoskeletal system, and so on. It is found in high expression levels both during bone development and fracture healing^14,16–21^.

Our previous research has confirmed that the Notch signal activated by canonical membrane protein ligand DLL1 can promote bone morphogenetic protein (BMP) (BMP2, 4, 6, 7, and 9) induced osteogenic differentiation of mesenchymal stem cells (MSCs) by affecting BMP signaling^22–24^. However, as a non-canonical secreted ligand of the Notch signaling pathway, how does CCN3 affect the osteogenic differentiation of MSCs? How does it coordinate with cell membrane ligand DLL1 to influence the osteogenic differentiation of MSCs? These are the primary coverage of our research.

Mouse embryonic fibroblasts (MEFs) are multipotent progenitor cells with the capacity of differentiating into tissues of both mesenchymal and non-mesenchymal origin^25^. MEFs have the characteristics of MSCs in vitro and in vivo^25^ and are considered to be an important cell line for MSC-related experiments^26–28^. So, in this study, we used the overexpressing CCN3 adenoviruses (Ad-CCN3) and small interfering CCN3 adenoviruses (Ad-siCCN3) to upregulate and downregulate CCN3 levels, respectively, in MEFs and explored the effects and mechanisms of CCN3 on osteoblastic differentiation of MEFs, so as to provide theoretical basis for the clinical application of CCN3-related proteins and factors in bone tissue engineering. Our results showed that CCN3 significantly inhibited the osteogenic differentiation of MEFs mainly through the inhibition of BMP signaling and the mutual inhibition with DLL1, so as to inhibit the expression of Hey1.

## Results

### The basic expression of CCN3 in MEFs

**Fig. 1:**
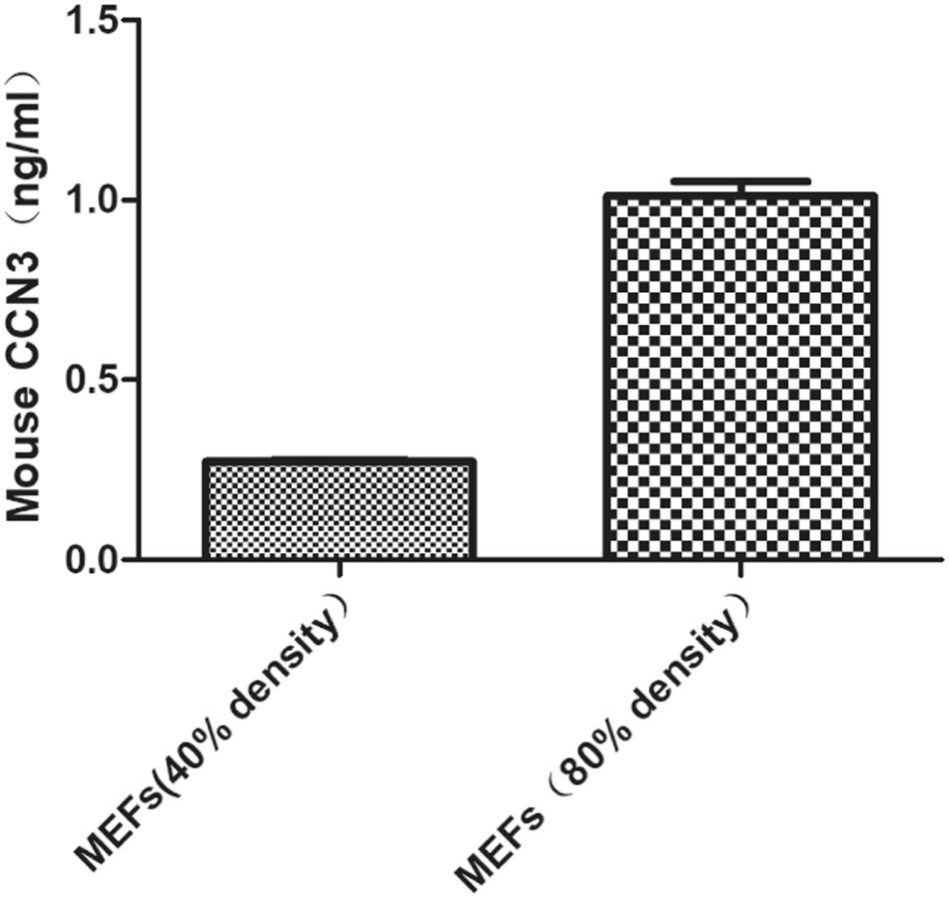
The basic expression of CCN3 in MEFs. ELISA assays to determine the CCN3 protein in cell culture supernatants. The data are shown as mean ± SD for three separate experiments

In order to detect whether the basic expression level of CCN3 in MEFs is very low, we used quantificational real-time polymerase chain reaction (qRT-PCR) and enzyme-linked immunosorbent assay (ELISA). The results of qRT-PCR showed that when the number of MEFs reached 5.8 × 10^5^ (at a density of 80%), the average cycle threshold (Ct) value of the CCN3 expression was 22.42. The results of ELISA assay showed that, when the number of MEFs reached 2.8 × 10^5^ (at a density of 40%), the concentration of CCN3 in the supernatant of cell culture was 0.27 ng/mL, while the concentration of CCN3 in the supernatant of cell culture was 1.01 ng/mL when the number of MEFs reached 5.8 × 10^5^ (at a density of 80%) (Fig. 1). These results indicated that CCN3 is expressed at low levels in MEFs.

### CCN3 inhibits early and late osteogenic differentiation of MEFs

**Fig. 2:**
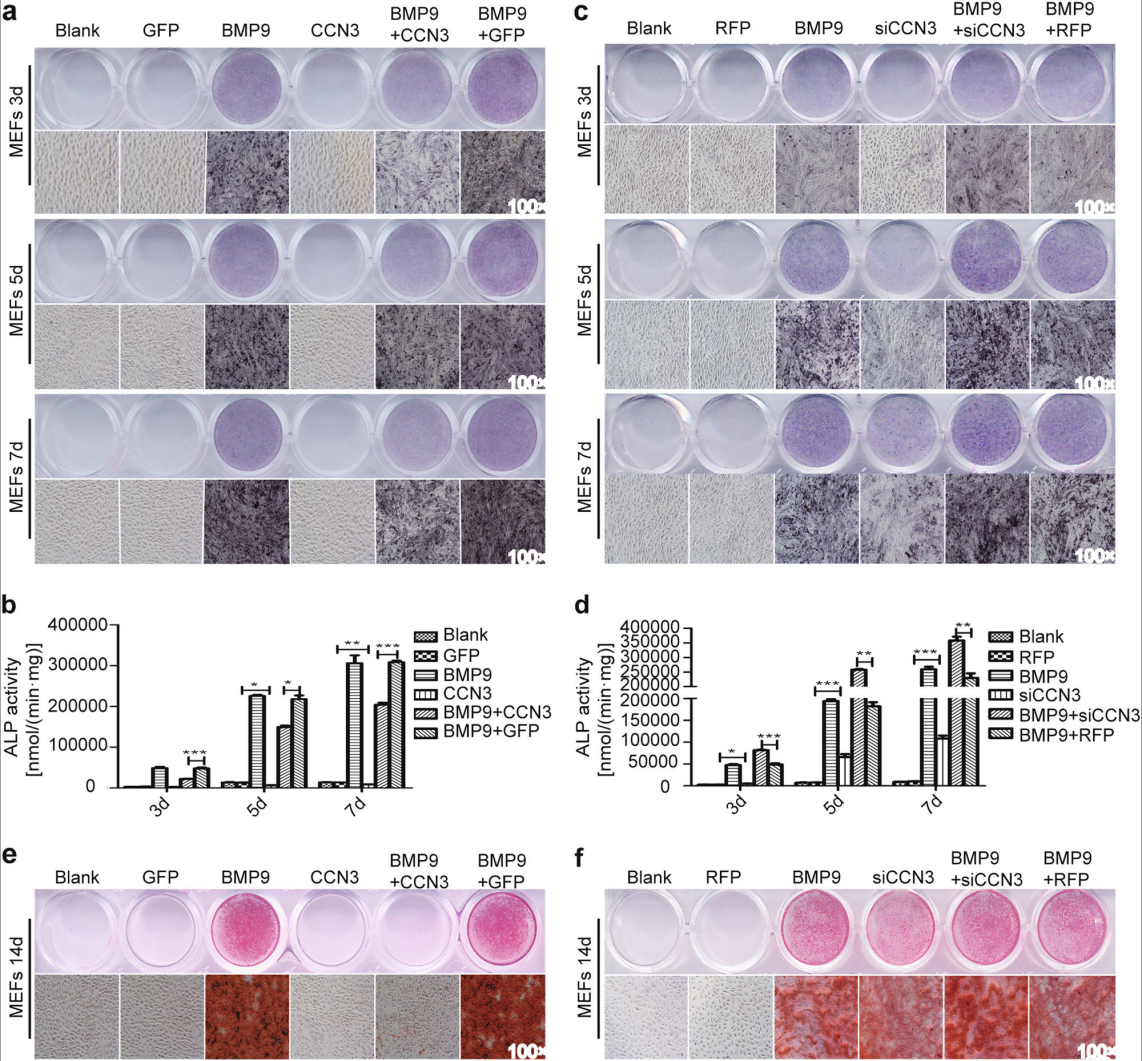
CCN3 inhibits early and late osteogenic differentiation of MEFs. **a**–**d** ALP staining assay (**a**, **c**) and quantitative assay (**b**, **d**) to determine the ALP activity under the treatment as shown at 3, 5, and 7 days postinfection (magnification ×100). **e**, **f** Alizarin Red S staining assay was used to assess matrix mineralization under the treatment as shown at 14 days postinfection (magnification ×100). The data are shown as mean ± SD for three separate experiments. *P < 0.05, **P < 0.01, ***P < 0.001

We sought to explore whether CCN3 had any effect on early and late osteogenic indicators or not. Alkaline phosphatase (ALP) assays was used to detect the changes of ALP activity, one of the early osteogenic indicators, and Alizarin Red S staining was used to detect the changes of calcium deposition, one of the late osteogenic indicators. We examined the effectiveness of Ad-CCN3 (Fig. S1a–c) and Ad-siCCN3 (Fig. S1d–f) by qRT-RCR and western blot. Ad-GFP and Ad-RFP were used as vector controls for these two adenoviruses, respectively. Therefore, Ad-CCN3 and Ad-siCCN3 were used to upregulate and downregulate CCN3 levels, respectively, in this study. The results showed that ALP activity and calcium deposition were obviously reduced in the BMP9+CCN3 group compared to BMP9+GFP group (Fig. 2a, b, e); whereas compared with the BMP9+RFP group, these indicators were increased significantly in the BMP9+siCCN3 group. The ALP activity and calcium deposition levels in siCCN3 group were also higher than that in the RFP control group (Fig. 2c, d, f). These results indicated that CCN3 could significantly inhibit the early and late osteogenic differentiation of MEFs.

### CCN3 inhibits the expression of osteogenic-related factors of MEFs

**Fig. 3:**
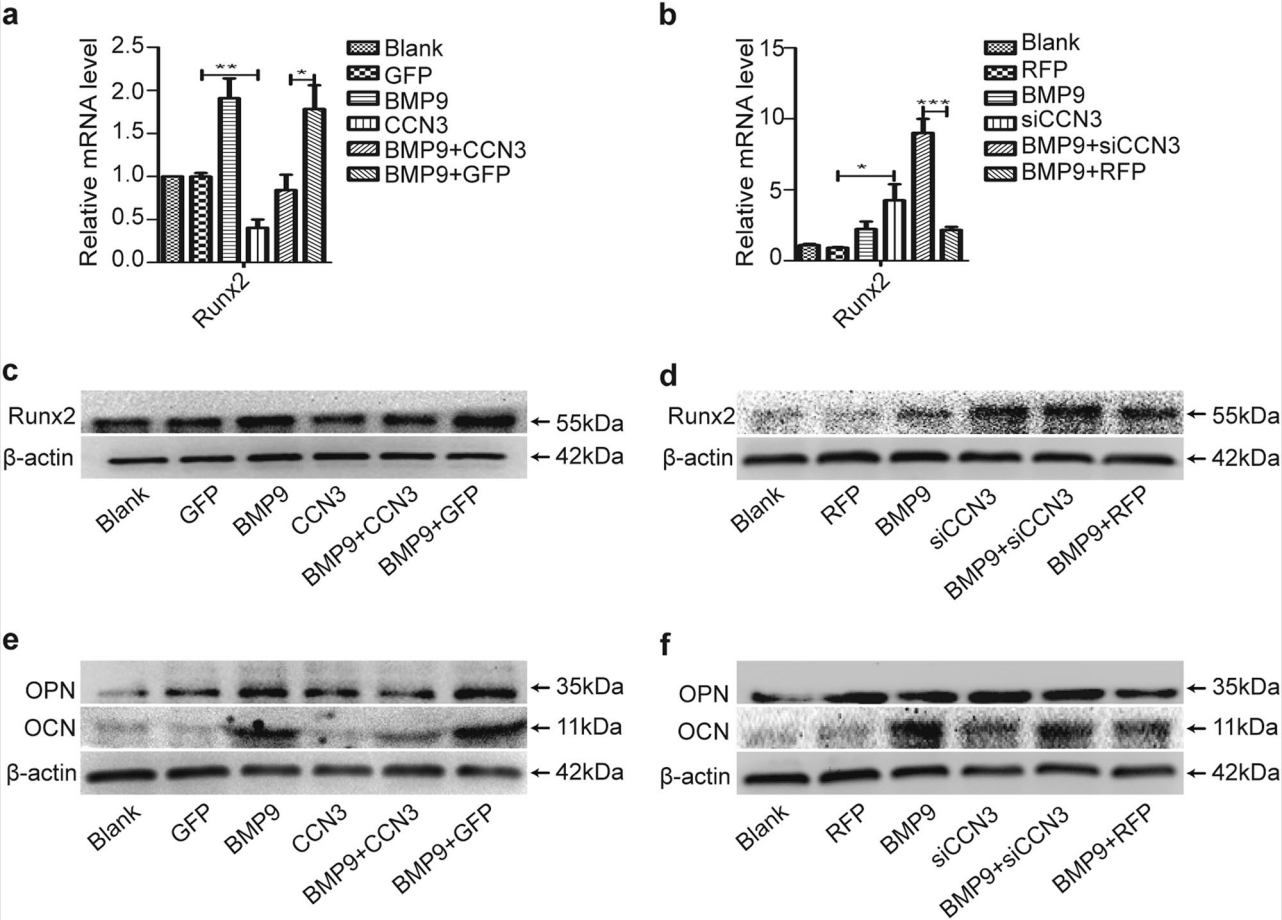
CCN3 inhibits the expression of osteogenic-related factors of MEFs. **a**, **c** qRT-PCR (**a**) and western blot (**c**) were adopted to detect the effects of CCN3 on the expression of Runx2 at 3 days posttreatment. **b**, **d** qRT-PCR (**b**) and western blot (**d**) were adopted to detect the effects of siCCN3 on the expression of Runx2 at 3 days posttreatment. **e**, **f** Western blot to determine the effects of CCN3 (**e**) and siCCN3 (**f**) on the expression of OPN and OCN at 7 days posttreatment. β-Actin was used as a loading control. The data are shown as mean ± SD for three separate experiments. *P < 0.05. **P < 0.01, ***P < 0.001

Runx2 is crucial in the process of osteogenesis. Runx2, osteopontin (OPN), and osteocalcin (OCN) are key indicators for osteogenic differentiation of MSCs. Thus we investigated the effects of CCN3 on the expression of Runx2, OPN, and OCN. Our results revealed that the expression levels of Runx2, OPN, and OCN in the BMP9+CCN3 group was significantly downregulated compared to the BMP9+GFP group (Fig. 3a, c, e); oppositely, the expression levels of these factors were increased obviously in the BMP9+siCCN3 group, as compared with the BMP9+RFP group (Fig. 3b, d, f). Additionally, the expression levels of these factors were also lower in the CCN3 group (Fig. 3a, c, e) and higher in the siCCN3 group (Fig. 3b, d, f) than in the vector control group. Taken together, these results indicated that CCN3 exerts a negative regulatory effect on the expression of osteogenic-related factors.

### CCN3 suppresses the subcutaneous ectopic osteogenesis of MEFs in nude mice

**Fig. 4:**
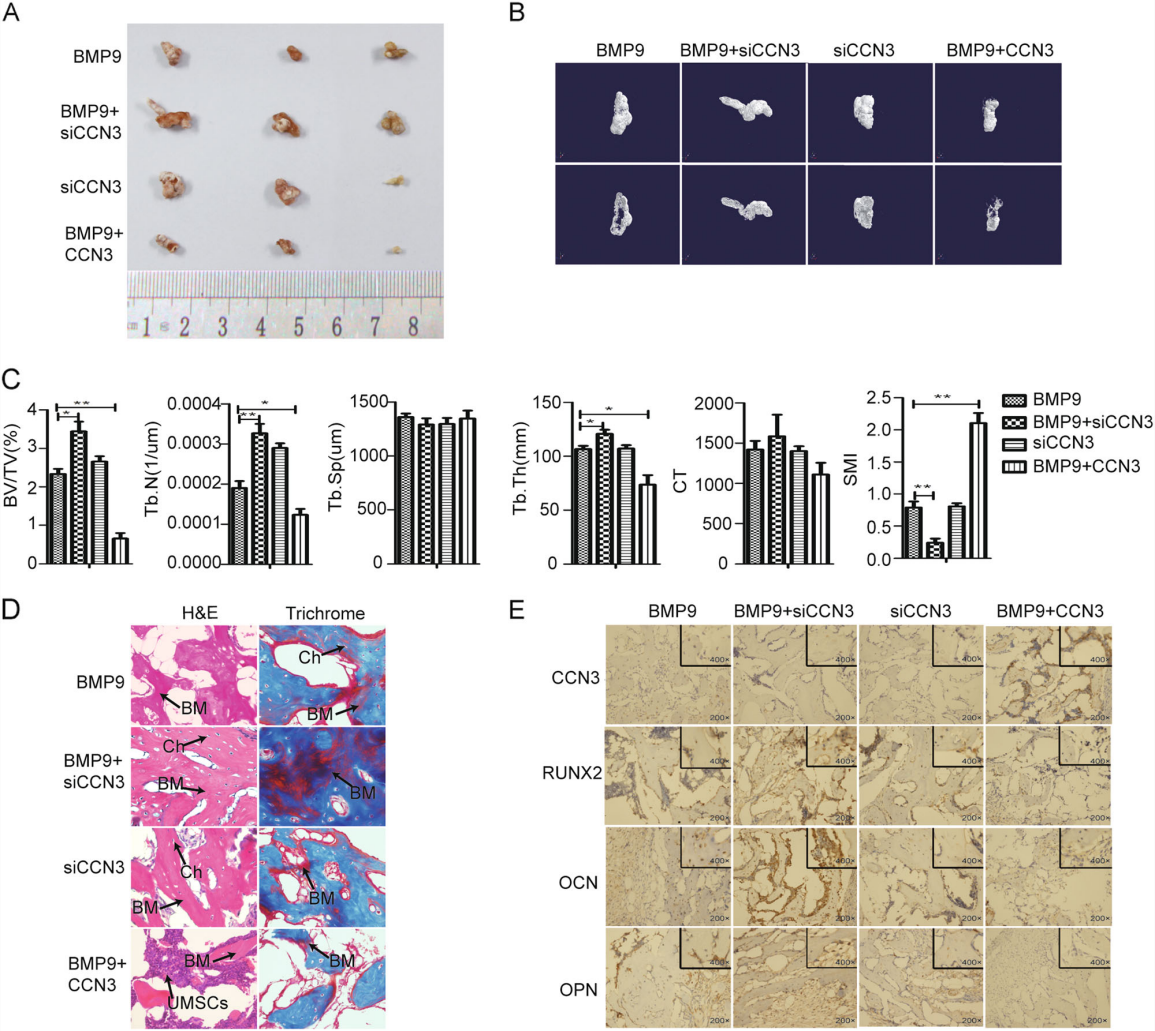
CCN3 suppresses the subcutaneous ectopic osteogenesis of MEFs in nude mice. **a** The general observation of the subcutaneous mass of ectopic osteogenesis in nude mice. **b** Subcutaneous osteoblast mass for micro-CT scanning to get a representative reconstructed 3D image, scaling 1 mm. **c** Quantitative analysis of bone tissue and the values of BV/TV, Tb.N, Tb.Sp, Tb.Th, CT, and SMI were analyzed. **d** H&E staining and Masson’s Trichrome staining to detect the formation of trabecular bone and bone matrix under the treatment as shown (magnification ×400). **e** Immunohistochemistry to verify the effects of CCN3 and siCCN3 on the expression of osteogenic-related markers (magnification ×200, ×400). The data are shown as mean ± SD for three separate experiments. *P < 0.05, **P < 0.01, ***P < 0.001

Based on the conclusion that CCN3 inhibited osteogenic differentiation of MEFs in vitro, we further confirmed it by subcutaneous ectopic osteogenesis in nude mice. The general observation and the results of micro-CT three-dimensional (3D) reconstruction showed that, compared with the BMP9 group, the volume of osteogenic masses was decreased in the BMP9+CCN3 group, while increased in the BMP9+siCCN3 group. The siCCN3 group also showed a strong ability to promote the formation of osteogenic masses (Fig. 4a, b). Further quantitative analysis of bone histomorphology revealed that the values of bone volume/total volume (BV/TV), trabecular number (Tb.N), and trabecular thickness (Tb.Th) were decreased, and the value of structural model index (SMI) (in cancellous bone, the SMI values of ideal rod trabecular bone and ideal plate trabecular bone are defined as 3 and 0, respectively) was increased in the BMP9+CCN3 group, as compared with the BMP9 group. While there were no significant differences in trabecular spacing (Tb.Sp) and CT (a relative value to identify bone density, the CT values of air and water are usually specified as −1000 and 0, respectively) values between the BMP9 group and the BMP9+CCN3 group. The results of the BMP9+siCCN3 group were opposite to those of the BMP9+CCN3 group (Fig. 4c). Additionally, histologic analysis revealed that, compared with the BMP9 group, the number and quality of trabecular bone, as well as the formation of bone matrix (red) in the BMP9+CCN3 group were decreased, while the number of trabecular bone and the formation of bone matrix in the BMP9+siCCN3 group was increased significantly, and the trabecular bone structure was more complete (Fig. 4d). Further immunohistochemical results showed that the expression levels of Runx2, OCN, and OPN were downregulated in the BMP9+CCN3 group, and upregulated in the BMP9+siCCN3 group, as compared with the BMP9 group (Fig. 4e). In summary, these data suggested that CCN3 exerts a negative regulatory effect on osteogenic differentiation of MEFs in vitro and in vivo.

### CCN3 restrains BMP9 signaling pathway

**Fig. 5:**
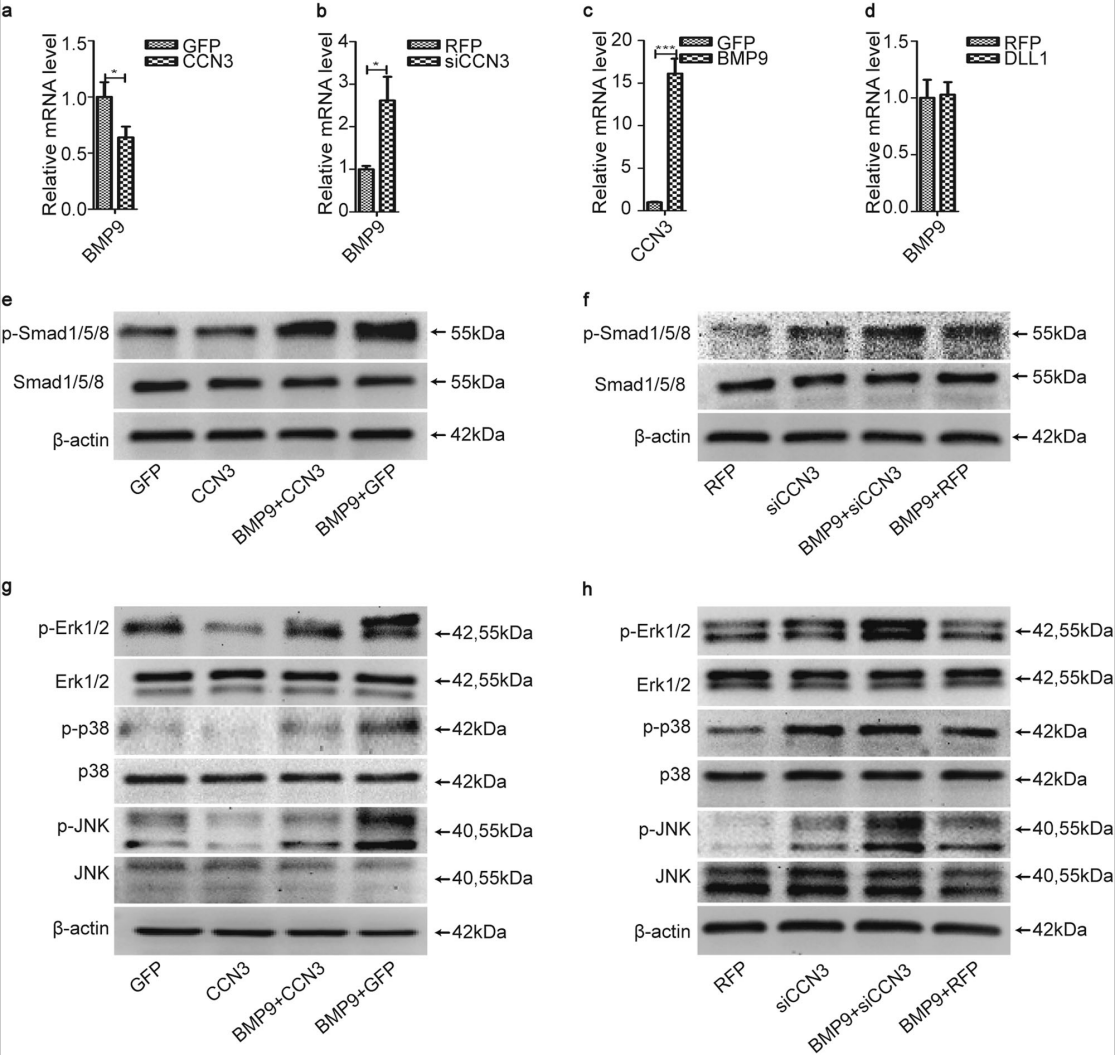
CCN3 restrains BMP9 signaling pathway. **a**, **b**, **d** qRT-PCR was adopted to detect the effects of CCN3 (**a**), siCCN3 (**b**), and DLL1 (**d**) on the mRNA level of BMP9 at 3 days posttreatment. **c** qRT-PCR to detect the effects of BMP9 on the mRNA level of CCN3 at 3 days posttreatment. **e**–**h** Western blot to determine the expression of p-Smad1/5/8, Smad1/5/8, p- Erk1/2, Erk1/2, p-p38, p38, p-JNK, and JNK under the treatment as shown at 3 days postinfection. The data are shown as mean ± SD for three separate experiments. *P < 0.05, **P < 0.01, ***P < 0.001

Our previous studies have confirmed that Notch signaling promoted osteogenic differentiation of MSCs induced by BMPs (BMP2, 4, 6, 7, and 9) through BMP signaling pathway^22–24^. Mitogen-activated protein kinases (MAPKs) are key branch of non-Smad BMP pathway. Zhao had demonstrated that BMP9 was able to activate extracellular signal–regulated kinase 1/2 (ERK1/2) and p38 MAPKs in mycobacterial phagosomal compartments and MAPKs (p38, ERK1/2, and c-Jun N-terminal kinases (JNKs)) play positive roles in BMP2-induced osteogenic differentiation^29^. Then does CCN3 also exert any impact on this signaling pathway? We further examined the effects of CCN3 on canonical and non-canonical BMP9 signaling pathways. The results of qRT-PCR showed that CCN3 downregulated the mRNA level of BMP9 predominantly (Fig. 5a) and siCCN3 played an opposite role (Fig. 5b). However, BMP9 upregulated the mRNA level of CCN3 (Fig. 5c), and the ELISA assay also showed that BMP9 upregulated the protein level of CCN3 in cell culture supernatants (Fig. S2), while DLL1 had no obvious effect on the mRNA level of BMP9 (Fig. 5d). The results of western blot revealed that the expression levels of p-Smad1/5/8, p-Erk1/2, p-p38, and p-JNK were lower in the CCN3 group (Fig. 5e, g) and higher in the siCCN3 group (Fig. 5f, h) than in the vector control group. Additionally, the expression levels of these proteins in the BMP9+CCN3 group were also significantly decreased compared to the BMP9+GFP group (Fig. 5e, g) and increased obviously in the BMP9+siCCN3 group, as compared with the BMP9+RFP group (Fig. 5f, h). Taken together, CCN3 significantly inhibited the phosphorylation of Smad1/5/8, Erk1/2, p38, and JNK in BMP9/Smad and BMP9/MAPK pathways, and we concluded that CCN3 inhibits osteogenic differentiation of MEFs by inhibiting the expression of BMP9 and then inhibits canonical BMP9/Smad signaling pathway and non-canonical BMP9/MAPK signaling pathway.

### CCN3 suppresses osteogenic differentiation of MEFs by inhibiting DLL1

We have confirmed that both non-canonical ligand CCN3 (secretory protein) and canonical ligand DLL1 (membrane protein) of Notch signaling pathway affected osteogenic differentiation of MEFs by regulating BMP signaling, and CCN3 inhibited osteogenic differentiation while DLL1 promoted osteogenic differentiation. We hypothesized that there was a competitive relationship between CCN3 and DLL1, and they coordinate with each other to regulate osteogenic differentiation of MEFs.

**Fig. 6:**
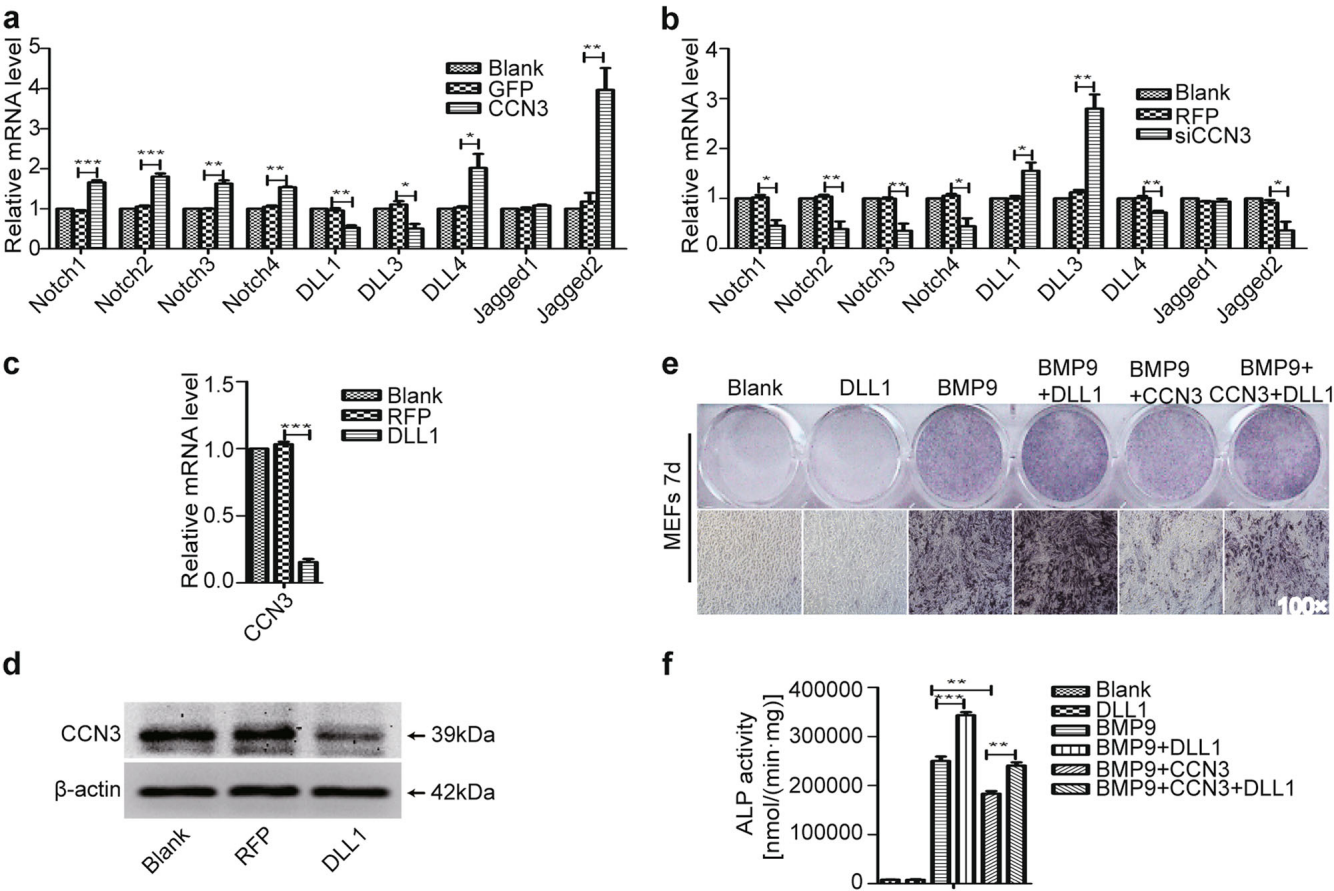
CCN3 suppresses osteogenic differentiation of MEFs by inhibiting DLL1. **a**, **b** qRT-PCR was adopted to detect the effects of CCN3 (**a**) and siCCN3 (**b**) on the mRNA levels of the canonical receptors and ligands of Notch signaling pathway at three days posttreatment. **c**, **d** qRT-PCR (**c**) and western blot (**d**) were used to determine the effects of DLL1 on the expression of CCN3 at 3 days posttreatment. **e**, **f** ALP staining assay (**e**) and quantitative assay (**f**) to determine the ALP activity under the treatment as shown at 7 days postinfection (magnification ×100). The data are shown as mean ± SD for three separate experiments. *P < 0.05, **P < 0.01, ***P < 0.001

To confirm this hypothesis, we used qRT-PCR to determine the mRNA levels of the canonical receptors and ligands of Notch signaling pathway. The results showed that the mRNA levels of all canonical receptors were higher in the CCN3 group than in the GFP group, prompting the activation effect of CCN3 on Notch signaling pathway. Conversely, lower mRNA levels of the ligands such as DLL1 and DLL3 were found in the CCN3 group, as compared with the GFP group (Fig. 6a); siCCN3 played an opposite role during this process (Fig. 6b). Additionally, we examined the effects of DLL1 on the expression of CCN3. The results of qRT-PCR and western blot showed that the expression level of CCN3 was decreased in the DLL1 group, as compared with the RFP group (Fig. 6c, d). We further examined the role of DLL1 in the process of CCN3 inhibiting osteogenic differentiation by ALP staining and quantitative assays. Our results showed that the activity of ALP in the BMP9+CCN3+DLL1 group was significantly higher than that in the BMP9+CCN3 group, suggesting that DLL1 could partially reverse the inhibitory effect of CCN3 on BMP9-induced osteogenic differentiation of MEFs (Fig. 6e, f). Collectively, these data suggested that there is a mutual inhibition relationship between CCN3 and DLL1, and CCN3 may inhibit osteogenic differentiation by inhibiting the expression of DLL1.

### CCN3 restrains osteogenic differentiation of MEFs by inhibiting Hey1

**Fig. 7:**
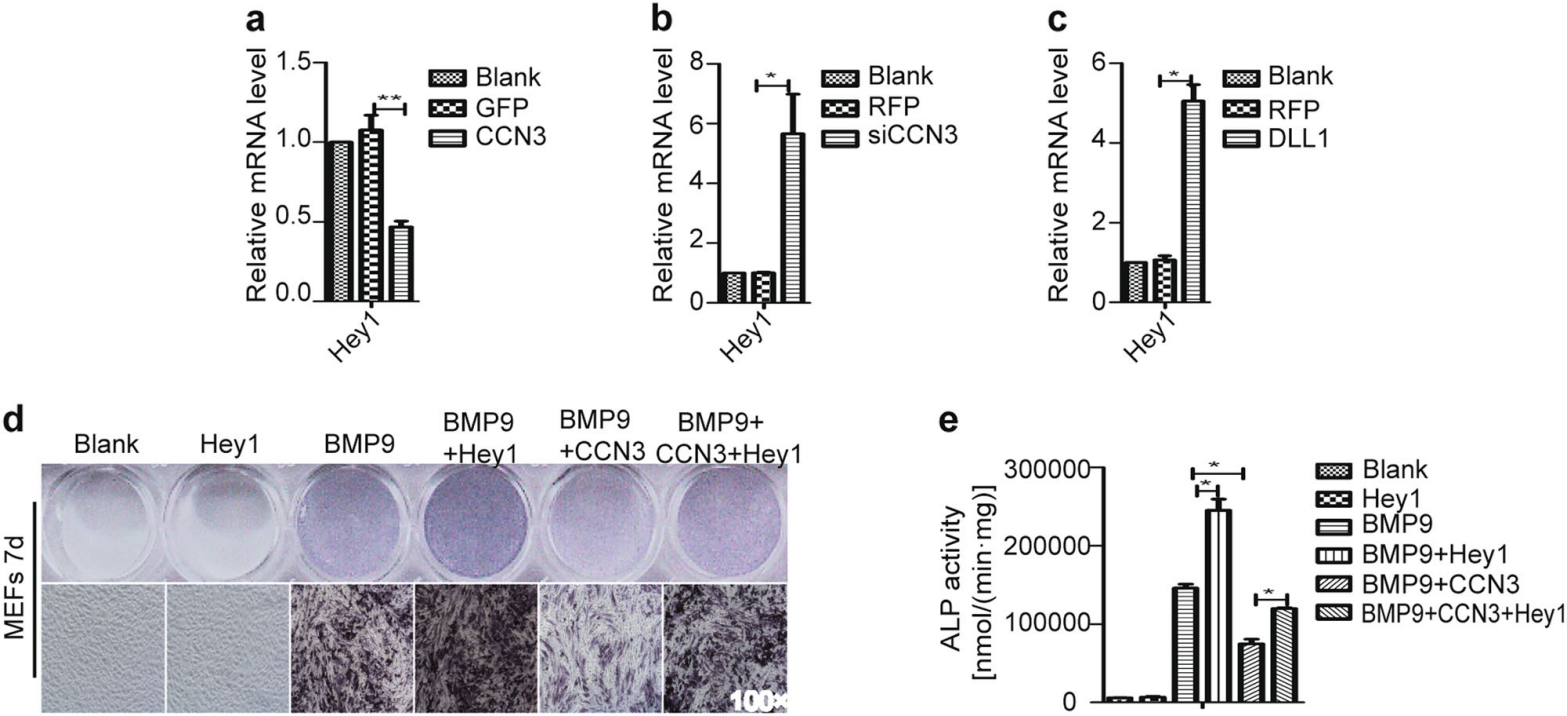
CCN3 restrains osteogenic differentiation of MEFs by inhibiting Hey1. **a**–**c** qRT-PCR was employed to measure the effects of CCN3 (**a**), siCCN3 (**b**), and DLL1 (**c**) on the mRNA level of Hey1 at 3 days posttreatment. **d**, **e** ALP staining assay (**d**) and quantitative assay (**e**) to determine the ALP activity under the treatment as shown at 7 days postinfection (magnification ×100). The data are shown as mean ± SD for three separate experiments. *P < 0.05, **P < 0.01, ***P < 0.001

Sharff^30^ had demonstrated that Hey1 played an indispensable role in BMP9-induced osteogenic differentiation of MSCs. Thus we investigated the role Hey1 played in the process of CCN3-induced inhibition of osteogenic differentiation of MSCs. First, we found that, for the expression of Hey1, CCN3 exerted a significant negative regulatory effect (Fig. 7a), while siCCN3, as well as DLL1, exerted a positive regulatory effect (Fig. 7b, c). Additionally, the results of ALP staining and quantitative assays revealed that the ALP activity level in the Hey1 group was similar to that of the blank control group, whereas increased significantly in the BMP9+Hey1 group, as compared with the BMP9 group. Furthermore, we also found that the activity of ALP in the BMP9+CCN3+Hey1 group was significantly higher than that in the BMP9+CCN3 group (Fig. 7d, e). These results suggested that Hey1 alone could not induce osteogenic differentiation but could promote BMP9-induced osteogenic differentiation. Furthermore, Hey1 could partially reverse the inhibitory effect of CCN3 on BMP9-induced osteogenic differentiation of MEFs. In summary, we conclude that Hey1 plays an important role in BMP9-induced osteogenic differentiation of MEFs, and CCN3 might affect the osteogenic differentiation of MEFs through or partially through Hey1.

### CCN3 inhibits subcutaneous ectopic osteogenesis of MEFs in nude mice by suppressing Hey1

**Fig. 8:**
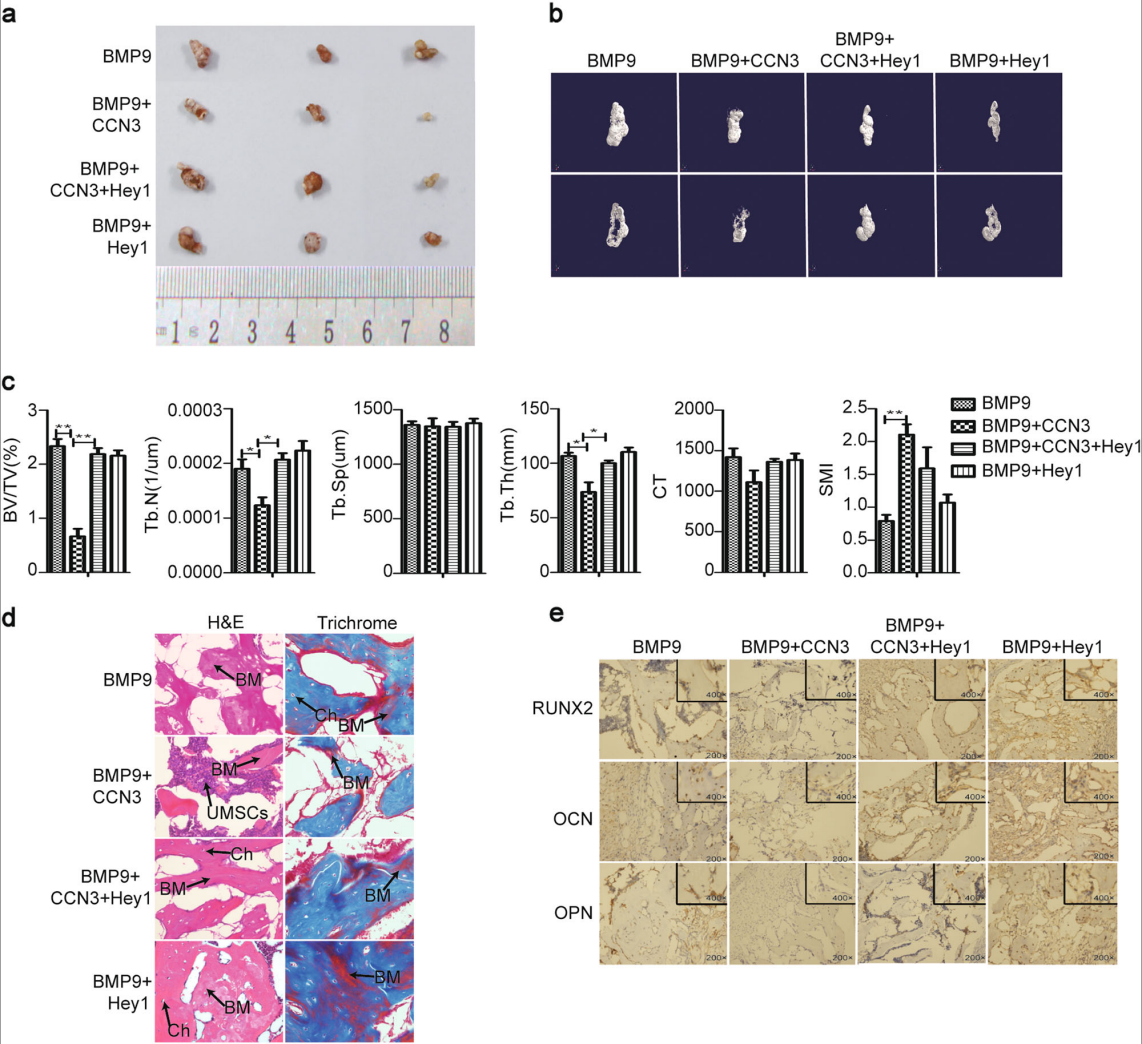
CCN3 inhibits subcutaneous ectopic osteogenesis of MEFs in nude mice by suppressing Hey1. **a** The general observation of the subcutaneous mass of ectopic osteogenesis in nude mice. **b** Subcutaneous osteoblast mass for micro-CT scanning to get a representative reconstructed 3D image, scaling 1 mm. **c** Quantitative analysis of bone tissue and the values of BV/TV, Tb.N, Tb.Sp, Tb.Th, CT, and SMI were analyzed. **d** H&E staining and Masson’s Trichrome staining to detect the formation of trabecular bone and bone matrix under the treatment as shown (magnification ×400). **e** Immunohistochemistry to determine the expression of osteogenic-related markers under the treatment as shown (magnification ×200, ×400). The data re shown as mean ± SD for three separate experiments. *P < 0.05, **P < 0.01, ***P < 0.001

We further tested the effect of Hey1 on CCN3-inhibited subcutaneous ectopic osteogenesis of MEFs in nude mice. The general observation and the results of micro-CT 3D reconstruction showed that the volume of osteogenic masses was increased in the BMP9+CCN3+Hey1 group, as compared with the BMP9+CCN3 group (Fig. 8a, b). Further quantitative analysis of bone histomorphology revealed that compared with the BMP9+CCN3 group, the values of BV/TV, Tb.N, and Tb.Th were increased in the BMP9+CCN3+Hey1 group, while there was no significant differences in Tb.Sp, CT, and SMI values between these two groups (Fig. 8c). Additionally, histologic analysis revealed that, compared with the BMP9+CCN3 group, the number and quality of trabecular bone, as well as the formation of bone matrix (red) in the BMP9+CCN3+Hey1 group were increased significantly, and the trabecular bone structure was more complete (Fig. 8d). Further immunohistochemical results showed that the expression levels of Runx2, OCN, and OPN were upregulated in the BMP9+CCN3+Hey1 group, as compared with the BMP9+CCN3 group (Fig. 8e). In summary, these results suggested that Hey1 plays an important role in the osteogenic differentiation of MEFs inhibited by CCN3, and it is a key intermediary factor affecting the osteogenic differentiation of MEFs.

## Discussion

CCN3/NOV is a member of the CCN family that was originally found in the study of finding the specific integration of myeloblastosis-associated virus type 1 in avian nephroblastoma^31^. It has been proved to play a crucial role in cell proliferation, differentiation, adhesion, migration, and apoptosis^21,32^. The current study on the role of CCN3 in osteogenic differentiation of MSCs has different perspectives. Some researchers found that CCN3 could inhibit osteogenic differentiation of MSCs: In vitro studies found by Minamizato et al.^33^ showed that CCN3 could significantly inhibit osteogenic differentiation of mouse embryonic osteoblast precursor cells (MC3T3-E1), which was consistent with the results of Katsube and others^34–37^. Matsushita et al.^17^ upregulated and downregulated CCN3 levels in vivo and found that CCN3 had the ability to significantly inhibit osteogenic differentiation, which was similar to the results of Rydziel and others^36–39^. However, there are also some authors who found that CCN3 can promote osteogenic differentiation of MSCs: Tan et al.^40^ pointed out that CCN3 could promote the osteogenic differentiation of rat primary osteoblasts; in the process of studying odontogenic differentiation of dental pulp stem cells (DPSCs), which was similar to the biological pathway of osteogenic differentiation of MSCs, Wang et al.^41^ found that CCN3 could promote odontogenic differentiation of DPSCs in vitro and in vivo.

To investigate the exact role of CCN3 in osteogenic differentiation of MSCs, we used MEFs with MSC characteristics in this study, upregulated and downregulated CCN3 levels by Ad-CCN3 and Ad-siCCN3, and detected the effects of CCN3 on ALP activity (one of the early osteogenic indicators), calcium deposition (one of the late osteogenic indicators), expression of osteogenic-related factors, and subcutaneous ectopic osteogenesis of MEFs in nude mice through in vitro and in vivo experiments. Our results demonstrated that CCN3 significantly inhibited the early and late osteogenic differentiation of MEFs and the expression of osteogenic factors Runx2, OPN, and OCN. Meanwhile, CCN3 also inhibited the osteogenic differentiation of MEFs induced by BMP9. These results were consistent with the in vitro findings of Katsube and others^33–37^. Additionally, our in vivo experiments indicated that CCN3 could inhibit subcutaneous ectopic osteogenesis in nude mice, reduce the volume of osteogenic mass, and the values of BV/TV, Tb.N, and Tb.Th. Meanwhile, CCN3 also increased the value of SMI and decreased the maturation level of osteogenic differentiation of MEFs, which were in accordance with the results of Rydziel and others^17,36–39^. Taken together, through in vitro and in vivo experiments, we confirmed the inhibitory effect of CCN3 on osteogenic differentiation of MEFs, and we systematically and comprehensively discussed the possible mechanisms of CCN3 affecting the osteogenic differentiation of MEFs afterwards.

Recent studies have indicated that CCN3 inhibits the osteogenic differentiation mainly by binding and neutralizing BMP2^33–36,38^ or promoting the expression of gremlin, a BMP2 antagonist^42^. Simultaneously, CCN3 also exerts a regulatory effect on Notch and Wnt/β-Catenin signaling pathways^33–36,38^.

Since BMPs and Notch signaling pathway play an important role in osteogenic differentiation, and our previous studies have confirmed that BMP9 had a strong stimulative ability^43^, whereas Notch signaling alone exerted no effect but could significantly promote BMP9-induced osteogenic differentiation^24^ during this process, we hypothesized that CCN3 plays an inhibitory role by affecting BMP9 and Notch signaling pathway. We first examined the effect of CCN3 on BMP9 expression. The results of qRT-PCR showed that CCN3 could reduce the mRNA level of BMP9, which was different from the findings that CCN3 only had a direct/indirect inhibitory effect on BMP2 protein reported by Rydziel and others^33–36,38,42^. However, BMP9 could upregulate the expression of CCN3, which suggested that there may be some negative feedback between CCN3 and BMP9. Our further study showed that CCN3 could significantly inhibit the phosphorylation levels of Smad1/5/8, which was similar to that of Rydziel and others^33–36,38^. Additionally, CCN3 could also inhibit the phosphorylation levels of Erk1/2, p38, and JNK, which was different from the perspective pointed out by Rydziel et al.^36^ that CCN3 had an effect on BMP2/MAPK pathway, but mainly inhibited the phosphorylation levels of Erk1/2. In summary, we conclude that CCN3 inhibits the osteogenic differentiation of MEFs by inhibiting the gene expression of BMP9 and downregulating the BMP/Smad and BMP/MAPK signaling pathways.

We further explored the effects of CCN3 on the canonical ligands and receptors of Notch signaling pathway. Our results revealed that CCN3 could promote the expression of Notch1, Notch2, Notch3, and Notch4, suggesting the activation effect of CCN3 on Notch signaling pathway. However, we found that CCN3 could inhibit the expression of DLL1, and in turn, DLL1 inhibited the expression of CCN3. Since our previous studies have confirmed that the Notch signaling activated by DLL1 could promote BMP9-induced osteogenic differentiation of MSCs, it can be speculated that CCN3 inhibited BMP9-induced osteogenic differentiation by downregulating the DLL1 expression. Meanwhile, CCN3 promoted the expression of Notch receptors, suggesting that it may also exert some effects through binding to Notch receptors, and the specific mechanism still needs further study.

Our previous studies have confirmed that Hey1 plays an indispensable role in BMP9-induced osteogenic differentiation of MSCs. Hey1 is an important and direct target of BMP9 signaling and is upregulated at the immediate early stage of osteogenic differentiation. Silencing Hey1 expression reduced BMP9-stimulated osteogenic differentiation both in vitro and in vivo^30^. We further explored the exact role of Hey1 in the process of osteogenic differentiation of MEFs inhibited by CCN3. The results of qRT-PCR confirmed that CCN3 could significantly downregulate the mRNA level of Hey1, which was consistent with the research of Rydziel et al.^36^. Then we detected the effects of Hey1 on CCN3-inhibited osteogenic differentiation of MEFs by ALP staining and quantitative assays, micro-CT scanning, and analysis of subcutaneous bony masses in nude mice, hematoxylin& eosin (H&E) staining, Masson’s Trichrome staining, and immunohistochemical testing. Our results showed that Hey1 alone did not have the ability to induce osteogenic differentiation, which was similar to the results of Sharff et al.^30^ in BMP9-induced osteogenic differentiation. However, Hey1 significantly promoted BMP9-induced osteogenic differentiation, which was consistent with the results of Sharff et al.^30^ and Wang et al.^44^. Moreover, Hey1 could partially reverse the downregulation effect of CCN3 on BV/TV, Tb.N, and Tb.Th, inhibit the upregulation of SMI, and reverse the inhibitory effect of CCN3 on osteogenic differentiation of MEFs. These results suggested that CCN3 inhibits Hey1 through both BMP and Notch signaling pathways. Hey1 plays an important role in the osteogenic differentiation of MEFs inhibited by CCN3, and it is a key intermediary factor affecting the osteogenic differentiation of MEFs, but the mechanism needs further clarification.

As ligands of the Notch signaling pathway, DLL1 and CCN3 play different roles in osteogenic differentiation. CCN3 is a kind of secretory ligand, it also works when there are fewer cells; but DLL1 is a membrane ligand, only fuctions when the cell number is big enough to contact with each other. So we suspect that, when the amount of cells is little, CCN3 inhibits BMP signaling and Notch signaling protein ligand DLL1, thereby inhibiting the expression of Hey1, the common target gene of BMP and Notch signaling pathways, and inhibiting osteogenic differentiation. As the number of cells increases, DLL1 activates the Notch signaling pathway when cells are in contact with each other, thus regulating the expression of the target genes such as Hey1 and promoting osteogenic differentiation. In this process, the effect of CCN3 on cell proliferation and apoptosis needs further research and confirmation.

In summary, this study identified the inhibitory effect of CCN3 on osteogenic differentiation of MEFs and further revealed that the inhibitory effect of CCN3 is mainly through the inhibition of BMP signaling and the mutual inhibition with DLL1 (one of the Notch signaling membrane ligands), so as to inhibit the expression of Hey1, the target gene shared by BMP and Notch signaling pathways. However, there are also some researchers who have put forward such views that CCN3 inhibits osteogenic differentiation at high concentration^36^ while has promoted effect at low concentration (physiological level)^40^. In our study, only adenovirus were used to upregulate and downregulate CCN3 levels, but no dose–effect studies were performed. Moreover, the way that CCN3 regulates the expression of BMP9 and DLL1 remains unclear and needs further study.

## Materials and methods

### Cell cultures

MEFs were isolated from post-coitus day 12.5 mice, as described previously^30,45^, and maintained in complete Dulbecco’s modified Eagle’s medium (DMEM) (HyClone, Logan, UT, USA) complemented by 10% fetal bovine serum (FBS) (Gibco, Grand Island, NY, USA) and antibiotics (50 IU penicillin/mL and 50 µg streptomycin/mL, PAA). Cells were incubated at 37 °C in 5% CO_2_. Human colorectal cancer cells (HCT116) were obtained from the ATCC (Manassas, VA) and cultured in the same condition as MEFs. All experimental protocols were approved by the Ethics committee of Chongqing Medical University Approval.

### Reagents and adenoviruses

DMEM high sugar medium was purchased from Hyclone Corporation (USA); FBS was obtained from Gibco Corporation (USA); ALP staining kit and substrate, vitamin C, β-phosphoglycerol, and Alizarin Red S were ordered from Sigma Corporation (USA); Anti-β-actin antibodies was obtained from Biyuntian Company (Jiangsu, China); Anti-Runx2, anti-OPN, anti-OCN antibodies, and the secondary antibodies were ordered from Cell Signaling Technology (CST, USA); Anti-Smad1/5/8, anti-p38, anti-Erk1/2, and anti-JNK antibodies were obtained from Zhongshan Golden Bridge Biotechnology (Beijing, China); Anti- phospho-Smad1/5/8, anti-phospho-p38, anti-phospho-Erk1/2, and anti-phospho-JNK antibodies were ordered from Santa Cruz Corporation (USA). The recombinant adenoviruses including Ad-CCN3, Ad-siCCN3, Ad-GFP, Ad-RFP, Ad-BMP9, Ad-DLL1, and Ad-Hey1 were kindly provided by Dr Tong-Chuan He (University of Chicago Medical Center, Chicago, IL, USA). GFP and RFP were used as tags for tracking the viruses, and Ad-GFP and Ad-RFP were used as vector controls. The effectiveness of the above viruses was confirmed in our and Professor He’s previous experiments^30^.

### Conditioned medium preparation

Subconfluent HCT116 cells (in 100 cm^2^ cell culture dishes) were infected with an optimal titer of Ad-BMP9 or Ad-GFP control, exchanged the medium with DMEM medium (without serum and antibiotics) 4 h later, and collected the culture medium after 1 day and 2 days, respectively. The culture medium was centrifuged at 1000 rpm for 5 min and then placed at 4 °C for use.

### qRT-PCR

Total RNA was extracted from MEFs with Trizol reagents (Takara, Otsu, Japan) and reverse transcription was performed using the Takara Prime Script RT Reagent Kit. The first-strand cDNA products were further diluted five-fold and used as PCR templates. RT-PCR reactions were performed using a SYBR Premix Ex Taq Kit (Takara, Otsu, Japan) on a PCR machine (7500 Real-Time PCR Detection System, Applied Biosystems, Alameda, CA, USA). All samples were normalized with the expression level of GAPDH and primers used are listed in Table 1.

**Table 1:** Sequences of primers used for qRT-PCR (mouse)

Primer	Forward	Reversed
GAPDH	GGCTGCCCAGAACATCAT	ATGATGTTCTGGGCAGCC
Runx2	GGTGAAACTCTTGCCTCGTC	AGTCCCAACTTCCTGTGCT
CCN3	ACGGAGAGAAGTTTGAGCCG	AGCCACAGGTCCACTTTTCG
BMP9	CTGCCCTTCTTTGTTGTCTT	CCTTACACTCGTAGGCTTCATA
Hey1	GGCCTGCTTGGCTTTTCT	CCAAGTGCAGGCAAGGTC
Notch1	GGTGAACAATGTGGATGCTG	GCAACACTTTGGCAGTCTCA
Notch2	GAGGATGAGGCTTTGCTGTC	GTTCTGCCTGAGGAGGAGTG
Notch3	CTCTGTGGTGATGCTGGAGA	AATCAAGTCGCTCCACTGCT
Notch4	AATCGGAGGTTCTGGATGTG	GGGTTCCAGATTTCCTAGCC
Jag1	GGAAGTGGAGGAGGATGACA	GTCCAGTTCGGGTGTTTTGT
Jag2	TCCGAGTACGCTGTGATGAG	GGCTTCTTTGCATTCTTTGC
DLL1	CCGGCTGAAGCTACAGAAAC	AGCCCCAATGATGCTAACAG
DLL3	TCTACCTCCCCCTACCGACT	CCTGATGTGGTTGAGCAAAA
DLL4	CCTCTCGAACTTGGACTTGC	TGGAAATACAGATGCCCACA

### ALP assays

MEFs were inoculated (at a density of 30%) in 24-well plates for 6 h and then infected with specific adenoviruses, followed by induced osteogenesis by BMP9 conditioned medium for 3/5/7 days after the fluorescence appeared. ALP staining and quantitative assays were measured at the specified time points. For ALP staining, MEFs were fixed with 4% formaldehyde, and the prepared ALP dyeing solution (250 μL/well) was added afterwards. Plates were protected from light and the staining results could be observed after 20 min. For ALP quantitative assay, MEFs were lysed with cell lysis buffer for 10 min and centrifuged at 13,000 rpm for 5 min. In another EP tube, 20 μL of the prepared ALP substrate was added, and 5 μL of cell lysate was pipetted into the tube, mixed well, and left standing for 60 min. The results were tested on Hitachi 7060C Automatic Biochemical Analyzer. Each assay condition was performed in triplicate and the results were repeated in three independent experiments. ALP activity was normalized by total cellular protein concentrations among the samples.

### Matrix mineralization assay (Alizarin Red S staining)

MEFs were inoculated (at a density of 30%) in 24-well plates for 6 h and then infected with specific adenoviruses, followed by induced osteogenesis by BMP9 conditioned medium, Vitamin C, and β-phosphoglycerol for 14 days after the fluorescence appeared. Alizarin Red S staining was used for matrix mineralization assay. MEFs were fixed with 0.05% (v/v) glutaraldehyde for 10 min and the mineralized matrices were stained with 0.4% Alizarin Red S for 5 min, followed by extensive washing with ddH_2_O. The results were recorded under microscope and repeated in at least three independent experiments.

### ELISA assay

MEF cell culture supernatants were collected and cell culture media were centrifuged at 2000 × *g* for 10 min to remove debris. Supernatants were collected and diluted five-fold. Expression levels of CCN3 protein were assayed using specific ELISA kits according to the manufacturer’s instructions (Abcam, Cambridge, UK).

### Western blot analysis

MEFs were inoculated (at a density of 30%) in 100 cm^2^ cell culture dishes for 6 h and then infected with specific adenoviruses, followed by induced osteogenesis by Ad-BMP9 for 3 days after the fluorescence appeared. Cells were harvested and washed with cold phosphate-buffered saline at least three times. Proteins were extracted with cell lysis buffer (20 mM Tris, pH 7.4, 150 mM NaCl, 1% P40, and 1 mM EDTA) supplemented with proteinase inhibitor and phosphatase inhibitor. The cell lysates were separated by sodium dodecyl sulfate polyacrylamide gel electrophoresis and then transferred onto polyvinylidene fluoride membranes. The blots was blocked in 5% bovine serum albumin for 2 h at 37 °C, incubated in the primary antibody (diluted 1:1000) overnight at 4 °C, and then incubated in a secondary antibody-conjugated to horseradish peroxidase (diluted 1:5000; Zhongshan Golden Bridge Biotechnology, Beijing, China) for 1 h at 37 °C. Finally, the membrane was exposed with ECL (Thermo Scientific, Rockford, IL, USA).

### Stem cell implantation and ectopic ossification

MEFs were infected with specific adenoviruses and harvested for subcutaneous injection (5 × 10^6^ cells per injection) into the flanks of athymic nude (nu/nu) mice (4–6-week-old male, Harlan Sprague-Dawley) until the fluorescence could been seen. At 4 weeks after injection, animals were euthanized, and the bony masses were collected for micro-CT imaging and histologic evaluation.

### Micro-CT imaging analysis, H&E, and Masson’s Trichrome staining

Animals were euthanized at 4 weeks after injection and scanned using the Scanner software of Skyscan1174 X-Ray Microtomograph (Micro CT) (Bruker company, Belgian). N-Recon software was used for 3D image reconstruction and all image data analysis was performed using the CT-AN software. Retrieved bony masses were decalcified with EDTA and then processed for paraffin embedding. Serial sections of embedded tissue were collected for H&E staining, Masson’s Trichrome staining, and immunohistochemical detection.

### Statistical analysis

Statistical analysis was performed by the SPSS 17.0 software. Each experiment was repeated three times independently. The data was expressed as mean (*x*) ± standard deviation (SD). One-way analysis of variance was used to compare multiple groups. The *t* test was performed between two groups. *P* < 0.05 was considered as statistically significant.

## Electronic supplementary material

Supplementary fig 1

Supplementary fig 2

Supplementary figure legends
