# Supplementary figures and images for: CCN3 and DLL1 co-regulate osteogenic differentiation of mouse embryonic fibroblasts in a Hey1-dependent manner
Source: Cell Death Dis. 2018 Dec 11;9(12):1188. doi: 10.1038/s41419-018-1234-1 (PMC6289993; doi:10.1038/s41419-018-1234-1)

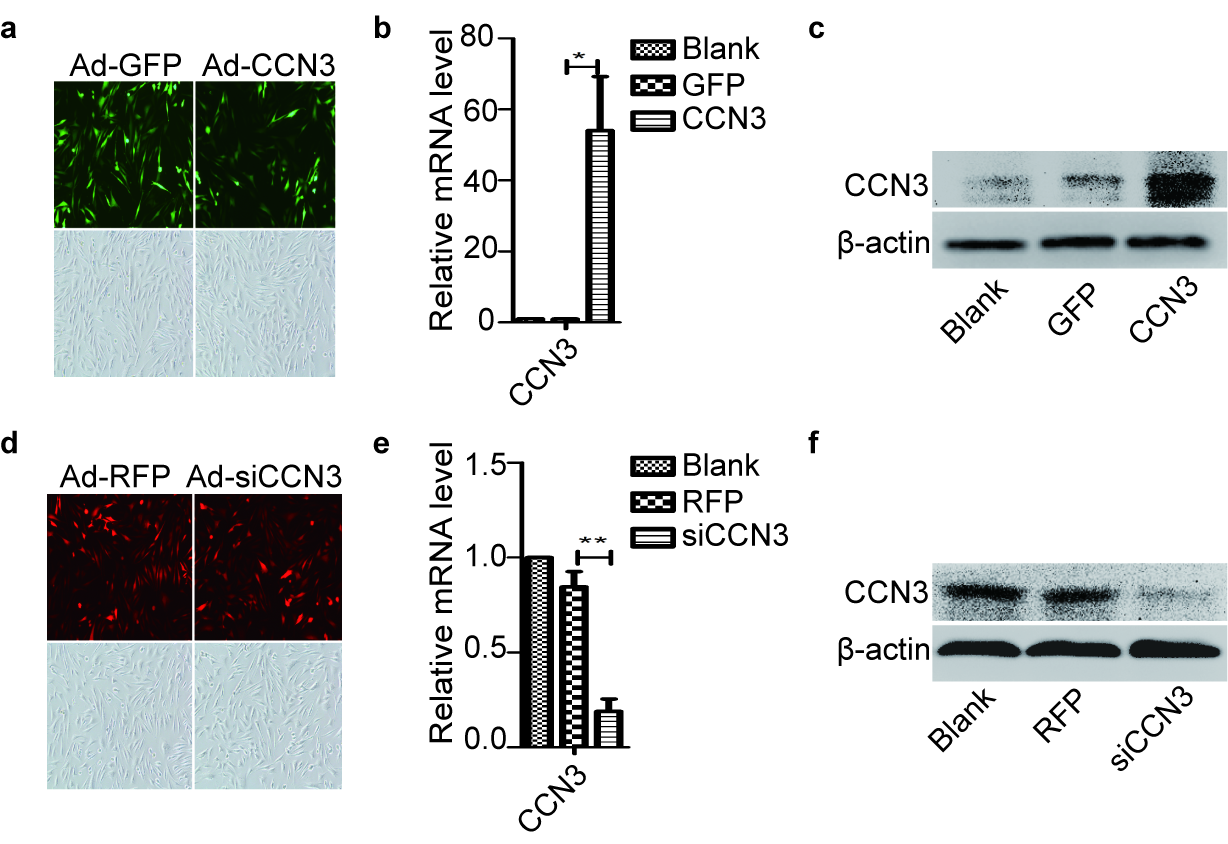

Supplement: Supplementary file 1 — Supplementary fig 1 [file 41419_2018_1234_MOESM1_ESM.tif]

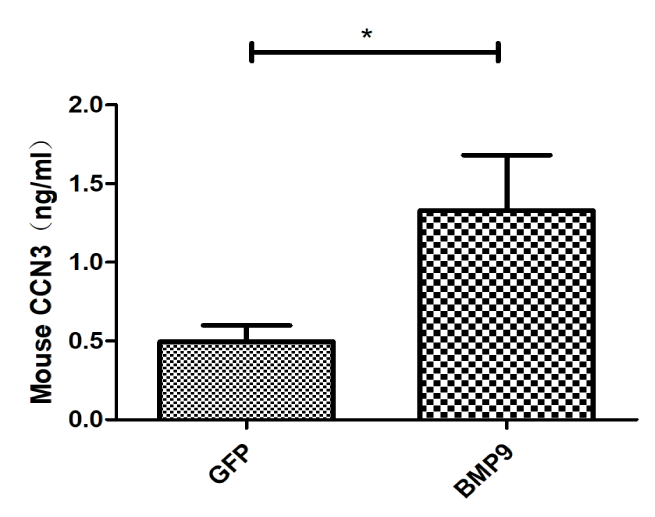

Supplement: Supplementary file 2 — Supplementary fig 2 [file 41419_2018_1234_MOESM2_ESM.tif]
