# Supplementary material for: CCN3 and DLL1 co-regulate osteogenic differentiation of mouse embryonic fibroblasts in a Hey1-dependent manner
Source: Cell Death Dis. 2018 Dec 11;9(12):1188. doi: 10.1038/s41419-018-1234-1 (PMC6289993; doi:10.1038/s41419-018-1234-1)
Supplement: Supplementary file 3 — Supplementary figure legends [file 41419_2018_1234_MOESM3_ESM.doc]

**Supplementary Figure Legends**

**Figure S1. Validation of the effectiveness of Ad-CCN3 and Ad-siCCN3. a** Fuorescent photos after Ad-CCN3 infected for 24 h (magnification ×100). **b, c** qRT-PCR (**b**) and Western blot (**c**) were used to detect the effects of Ad-CCN3 on the expression of CCN3 at 3 days post-treatment. **d** Fuorescent photos after AdsiCCN3 infected for 36 h (magnification ×100). **e, f** qRT-PCR (**e**) and Western blot (**f**) were adopted to detect the effects of Ad-siCCN3 on the expression of CCN3 at 3 days post-treatment. For western blot, β-actin was used as a loading control. The data were shown as mean ± SD for 3 separate experiments. **P*< 0.05, ***P*< 0.01.

**Figure S2.** **BMP9** **promotes the expression of CCN3.** **a** ELISA assay to detect the expression of CCN3 protein. The data were shown as mean ± SD for 3 separate experiments, **P*< 0.05.
